# One-stage revision versus debridement, antibiotics, and implant retention (DAIR) for acute prosthetic knee infection: an exploratory cohort study

**DOI:** 10.1007/s00402-023-04891-1

**Published:** 2023-04-27

**Authors:** Charles Ebuka Okafor, Son Nghiem, Joshua Byrnes

**Affiliations:** 1 Centre for Applied Health Economics, School of Medicine, and Dentistry, Griffith University, 170 Kessels Road, Nathan, QLD 4111 Australia; 2 Menzies Health Institute, Griffith University, Queensland, Australia; 3 Department of Health Services, Research and Policy, Australian National University, Canberra, Australia

**Keywords:** Knee, Prosthetic joint infection (PJI), Acute postoperative PJI, Hematogenous infection, DAIR—debridement, antibiotic, and implant retention, One-stage revision

## Abstract

**Introduction:**

Studies have shown that debridement, antibiotics, and implant retention (DAIR) is an effective procedure for acute infection of total knee arthroplasty (TKA). This study aimed to explore DAIR and one-stage revision for homogenous cohorts with acute postoperative and acute hematogenous infection of TKA, without compelling indications to perform a staged revision.

**Materials and methods:**

This study was an exploratory analysis that used retrospective data from Queensland Health, Australia, for DAIR and one-stage revision of TKA between June 2010 and May 2017 (3-year average follow-up). The re-revision burden, mortality rate, and the cost of the interventions were explored. Costs were expressed in 2020 Australian dollars.

**Results:**

There were 15 (DAIR) and 142 (one-stage) patients with homogenous characteristics in the sample. The re-revision burden for DAIR was 20%, while for one-stage revision it was 12.68%. Two deaths were associated with a one-stage revision and no death was associated with DAIR. The total cost since the index revision of DAIR, $162,939, was higher than for one-stage revision $130,924 (*p* value = 0.501), due to higher re-revision burden.

**Conclusions:**

This study would suggest the use of one-stage revision over DAIR for acute postoperative and acute hematogenous infection of TKA. It suggests that there could be other potential criteria which have not been ascertained that need to be considered for optimal DAIR selection. The study indicates the need for more research and, of note, high-quality randomized controlled trials to provide a well-defined treatment protocol with high level of evidence to guide patient selection for DAIR.

**Supplementary Information:**

The online version contains supplementary material available at 10.1007/s00402-023-04891-1.

## Introduction

Prosthetic joint infection (PJI) is a major complication of total knee arthroplasty (TKA) and a leading cause of knee revision in Australia [1]. The management approach for a patient depends on several factors, including patient and diagnostic factors. For acute postoperative PJI and acute hematogenous infection of TKA, the recommended management approach is either debridement, antibiotics, and implant retention (DAIR) or one-stage septic revision [2]. Two-stage septic revision is mostly reserved for chronic PJI with or without methicillin-resistant *Staphylococcus aureus* (MRSA) PJI, multi-organism PJI, systemic sepsis, presence of comorbidities, culture-negative PJI, PJI by resistant organisms, and patients who are immunocompromised, while salvage procedures (resection arthroplasty, arthrodesis and amputation) are reserved for patients with persistent PJI who are unable to retain functional TKA or where there is a high risk of failure after revision or re-revision [2]. As DAIR procedure requires a less invasive procedure, has better functional knee score, minimal bone loss and soft tissue trauma, lower morbidity, and relatively lower cost compared to staged revision, it is usually an attractive procedure for many patients and surgeons in the case of acute PJI [3].

It remains unclear when to effectively perform DAIR. When the timing for DAIR is missed and other factors are not adequately weighed, its risk will outweigh its benefit. DAIR is commonly recommended for acute infection postoperative period within 4 weeks of surgery or acute haematogenous infection of TKA within 2 weeks of onset [3], when biofilm has not been formed. Some other criteria necessary for a successful DAIR include low virulence organisms, organisms sensitive to antibiotics, no component loosening, proper alignment, no osteomyelitis, and no sinus tract [2]. The duration of symptoms is also becoming an important consideration for DAIR [4]. Patients who are young and healthy with PJI after primary TKA are more likely to have successful DAIR [2]. Arthroscopic DAIR is rarely performed due to the high failure rate [2]; it has no role in the management of acute PJI and was not included in this study. The exchange of polyethylene and the duration of antibiotic therapy are necessary for a successful DAIR. These days, the duration of antibiotics following DAIR or one-stage septic revision is usually 6–12 weeks, but in some cases could extend up to 24 weeks [5].

Several studies have shown that DAIR is an effective procedure for early postoperative or acute PJI of the knee [6–8], but it is challenging to accurately interpret the results of most published DAIR results due to the heterogeneity of the described surgical technique and differences in patients and diagnostic factors.

As knee revision due to infection has been on the increase recently, the right patient selection for the different interventions for PJI is important. The right patient selection will ensure optimal quality of life for the patients, reduce the risk of re-revision, and minimize the excess cost associated with making the suboptimal treatment choice. Based on current treatment recommendations in the literature, this study aimed to explore the use of DAIR and one-stage revision for homogenous patients with acute postoperative PJI and acute hematogenous infection of TKA, without compelling indications to perform a staged revision. The outcome of this study will support decision-making in the management of acute PJI of the knee.

## Materials and methods

### Population and sample size

The study was conducted using Queensland Health data for patients who had acute knee revision(s) due to PJI or hematogenous infection after primary knee TKA between June 2010 and May 2017 in Queensland public hospitals. There was a total of 368 patients who had septic knee revision after primary TKA. After excluding patients with indications that would compel the performance of one-stage or two-stage septic revision, patients with chronic PJI, patients with methicillin-resistant *Staphylococcus aureus* (MRSA) PJI, multi-organism PJI, systemic sepsis, component(s) loosening, mal-alignment, sinus tract, comorbidities, culture-negative PJI, PJI by a resistant organism, arthroscopic DAIR, and patients who were immunocompromised [9], the final sample size for analysis was 157.

### Data set and requirement

Data were obtained from Queensland Hospital Admitted Patient Data Collection (QHAPDC), Queensland Hospital Non-Admitted Patient Data Collection (QHNAPDC), National Hospital Cost Data Collection (NHCDC), and iPharmacy, which were linked by the Queensland Health Statistical Service Branch (SSB). We used de-identified patient-level data. Identification of in-scope data was achieved using the International Statistical Classification of Diseases and Related Health Problems, Tenth Revision, Australian Modification (ICD-10-AM), and the 11th Edition of the Australian Classification of Health Interventions (ACHI). Index revisions performed by DAIR were identified by the 4,952,700 ACHI procedure code, which is the code applicable for knee DAIR in Australia and New Zealand. For one-stage septic revisions, identification of patients was achieved with the following ACHI codes: 4,951,500; 4,952,700; 4,953,000; 4,953,001; 4,953,300; 4,954,800; or 4,955,400 (See the supplementary file). As 4,952,700 was a common code for both DAIR and one-stage revision, DAIR was differentiated from one-stage revision with the aid of the diagnostic code Y831 (surgical operation with an implant of an artificial internal device), where the presence of Y831 indicates one-stage revision, while its absence indicates DAIR (which does not require implant exchange but polyethylene). Patients with compelling indications were identified using the ICD-10-AM codes and were excluded from the analysis. The supplementary file describes the compelling indications.

### Ethical approval

Ethical approval was granted by the Griffith University Human Research Ethics Committee with a reference number, Ref: 2020/409.

### Interventions and comparator

The interventions include DAIR and one-stage exchange septic revision for acute PJI and hemtogenous knee infection. DAIR was assessed as the base-case intervention, while one-stage septic revision was the comparator. DAIR revision involves washout, thorough debridement, antibiotics, and all components of implant retention, but polyethylene exchange. One-stage exchange revision involves open debridement of the infected knee, followed by immediate revision by removal and or reimplantation of all components.

### Costs of the interventions

The cost of hospitalization for DAIR and for one-stage septic revision events were explored from the healthcare provider perspective using bottom-up costing approach and compared for the homogenous cohorts. The associated additional costs after the index revisions were also estimated for an average of 3 years. The additional cost after the index revision was due to outpatient clinic visits, physiotherapy appointments, radiological examinations, post-surgical antibiotics, other allied health costs, and also the cost of re-revision or surgical intervention due to treatment failure.

The costs were estimated from the healthcare provider’s perspective. The costs include the direct medical and direct non-medical costs of the surgical procedure, intensive care units, hospital stays, medications, prostheses, medical equipment, and devices used, laboratory tests, radiology tests, physiotherapy visits, nursing fees, consultations, pharmacy service costs, and other allied health costs. All costs were expressed in 2020 Australian dollars (AUD).

### Outcomes of the interventions

The outcomes of DAIR and one-stage septic revision were measured as the re-revision burden and mortality rate. The number of re-revisions that occurred after the index revision by DAIR was compared to the re-revisions after the index revision by the one-stage procedure. The mortality risk was also compared between the two groups.

### Data analyses

An exploratory analysis of DAIR and one-stage was performed. Data analyses were performed using STATA 14 (StataCorp, College Station, USA). Descriptive statistics were used to analyze the study characteristics, costs, and outcomes. Results were expressed as a mean (with a 95% confidence interval) for continuous variables. Categorical variables were presented as percentages or frequencies. The Student’s *t* test was used to compare continuous variables, while Fisher’s exact test was used for categorical variables. Differences were considered statistically significant if the *p* values ≤ 0.05.

## Results

### Patients’ characteristics and health service utilization

After controlling for compelling indications to perform a staged revision, a total of 15 patients and 142 patients met the inclusion criteria for acute postoperative PJI for DAIR and one-stage septic revision, respectively. These patients were followed up for an average of 3 years after their index revision. Most revision procedures occurred within the age group of 65–69 years (19%) for DAIR and 70–74 years (20%) for one-stage revision. The majority of the patients were from major cities of Queensland (50%). 17.54% and 16.08% of the patients who had DAIR and one-stage revision, respectively, had private hospital insurance (*p* value = 0.629). The indigenous population who had knee revision were less than 3%, while the non-indigenous accounted for about 97%. Most revision operations (91%) were elective (*p* value < 0.001); The length of hospital stay (LOS) was similar between DAIR 16 (95% CI 14–19) days and one-stage revision, 15 (95% CI 14–16) days (*p* value = 0.896). No DAIR case required an intensive care unit (ICU) stay, but 3% of patients who had one-stage revision required ICU. Details of the patients’ characteristics are available in Table 1.

**Table 1:** Patients’ characteristics and health service utilization

Variables	DAIR, n (%)	One-stage revision, *n* (%)	*p* value^a^
Age group of patients (years)			< 0.001
05–59	2 (12.27)	27 (18.42)	
60–64	2 (15.79)	21 (14.12)	
65–69	3 (19.30)	29 (19.41)	
70–74	2 (10.53)	30 (20.39)	
75–79	3 (17.54)	20 (13.73)	
80–84	2 (14.04)	14 (9.02)	
≥ 85	1 (10.53)	8 (4.91)	
Sex			< 0.001
Female	9 (58.00)	58 (39.00)	
Male	6 (42.00)	91 (61.00)	
Private insurance status for admission			
Insured	2 (16.00)	24 (16.00)	< 0.001
Not insured	12 (79.00)	125 (84.00)	< 0.001
Not stated/unknown	1 (5.00)	0 (0.00)	n/a
Indigenous status			
Indigenous	1 (3.51)	4 (2.94)	0.001
Non-indigenous	14 (96.49)	145 (97.06)	< 0.001
Elective status			
Elective admission	7 (48.00)	87 (58.43)	< 0.001
Emergency admission	7 (48.00)	57 (38.04)	< 0.001
Not assigned	1 (4.00)	5 (3.53)	0.004
Revisions that used ICU	0 (0.00)	4 (2.55)	n/a
Length of hospital stay per revision	16 (14–19)	15 (14–16)	0.896

*DAIR* debridement, antibiotic, and implant retention, *ICU* intensive care unit

^a^DAIR versus one-stage revision

### Cost of DAIR and one-stage revision

We found the average treatment cost by DAIR, $50,974 (95% CI $43,201–$58,747), to be less expensive than one-stage revision, $53,410 (95% CI $49,587–$57,232), (*p* value = 0.684). However, all costs since after the index revision of DAIR, $162,939 (95% CI $119,974–$205,904), was more expensive than one-stage revision $130,924 (95% CI $114,948–$146,900), (*p* value = 0.501). The detailed costs are presented in Table 2.

**Table 2:** Direct costs of the interventions

Component	Mean (95% confidence interval) ($)		*p* value^a^ (DAIR vs one-stage revision)
Total direct cost	DAIR	One-stage revision	
Revision event	50,974 (43,201–58,747)	53,410 (49,587–57,232)	0.684
Index revision + follow-up^β^	162,939 (119,974–$205,904)	130,924 (114,948–$146,900)	0.501
Direct medical costs			
Total direct medical cost	43,534 (36,645–50,423)	46,098 (42,780–49,416)	0.622
Allied health	1374 (1001–1747)	1775 (1537–2014)	0.277
Critical care	1816 (0–3748)	1782 (871–2693)	0.981
Depreciation	441 (350–531)	452 (401–503)	0.888
Hotel	640 (488–793)	595 (545–645)	0.573
Imaging	755 (528–983)	698 (614–782)	0.668
Non-clinical salaries	1718 (1342–2094)	1816 (1619–2014)	0.750
Operating room	9433 (8231–10,634)	10,685 (9852–11,519)	0.331
Oncosts^§^	2368 (1925–2810)	2384 (2171–2597)	0.961
Pathology	1331 (1049–1612)	1688 (1509–1868)	0.199
Pharmacy	1694 (1172–2215)	1855 (1513–2197)	0.760
Prosthesis	6653 (3924–9382)	7376 (6463–8290)	0.622
Special procedure suites	70 (0–165)	121 (74–168)	0.482
Ward medical (specialist/officer)	4457 (3044–5869)	4223 (3785–4661)	0.742
Ward nursing	8990 (6964–11,016)	8646 (7819–9473)	0.793
Ward supplies	1794 (1150–2438)	1999 (1693–2305)	0.669
Direct non-medical costs			
Total direct non-medical cost	7440 (6209–8670)	7312 (6723–7900)	0.890
Allied health	310 (214–407)	387 (345–429)	0.248
Critical care	364 (0–807)	360 (180–540)	0.989
Depreciation	298 (135–460)	382 (295–468)	0.533
Hotel	32 (16–48)	32 (24–41)	0.966
Imaging	129 (95–163)	111 (95–126)	0.447
Non-clinical salaries	424 (226–622)	461 (374–548)	0.788
Operating room	1722 (1432–2012)	1543 (1416–1671)	0.374
Oncosts^§^	148 (78–218)	175 (142–209)	0.598
Patient travel subsidy	316 (117–535)	174 (22–326)	0.490
Pathology	133 (75–191)	117 (91–144)	0.698
Pharmacy	115 (80–150)	124 (109–140)	0.696
Special procedure suites	7 (0–16)	9 (3–16)	0.804
Ward medical (specialist/officer)	2659 (1693–3624)	2176 (1869–2482)	0.330
Ward nursing	554 (253–856)	682 (561–803)	0.503
Ward supplies	530 (368–691)	744 (631–857)	0.221

*DAIR* Debridement, antibiotics, and implant retention ^a^Two-sample Student’s *t* test

^§^The oncosts as used by Queensland health management includes superannuation, termination payment, lump-sum payment, fringe benefits tax, income tax expense, long service leave, annual leave, accrued days off, other leave, work cover premium (workers compensation), and recruitment costs

^β^Three-year follow-up

### Outcomes of DAIR and one-stage revision

Patients who had DAIR had a total of nine re-revisions, and the re-revision burden was 20.00%. Patients who had a one-stage revision had a total of 54 re-revisions, and the re-revision burden was 12.68%. There was no death associated with DAIR, but two deaths were associated with a one-stage revision.

## Discussion

This study explored the failure rate (proxied by re-revision burden and mortality) and the management cost of DAIR and one-stage revision based on current treatment recommendations in the literature. There was a higher re-revision burden with the use of DAIR for acute postoperative PJI and acute hematogenous infection following index revision, compared to one-stage revision. Also, the cost of DAIR plus its additional cost due to follow-up was higher than that for a one-stage revision. However, the cost of DAIR hospitalization alone (without follow-up cost) was lower than that for a one-stage revision, but the difference was not statistically significant. The primary reason for the higher follow-up cost with DAIR compared to a one-stage revision was due to its higher rate of failure and re-revision. The plausible reason for no observed death with DAIR was due to the smaller sample size compared to the one-stage group. Nonetheless, the result suggests that more deaths would be associated with one-stage septic revision compared to DAIR in the case of a larger cohort.

The findings from this study would suggest the use of one-stage revision over DAIR for acute PJI in patients without the compelling indications described earlier, but it is also indicative that further criteria need to be explored and considered to select candidates for DAIR. Generally, it is believed that if the criteria to select patients for DAIR are optimal, the rate of re-revision or re-revision burden after DAIR should not be higher, but rather similar to or lower than the rate of re-revision after a staged revision. It is also widely accepted that staged revision is not superior to DAIR. The key consideration remains that the patient selection has to be optimal. Optimal patient selection could reduce the re-revision burden for DAIR and the cost of DAIR to make the procedure more attractive compared to one-stage revision. Although this study was exploratory and did not measure the quality of life of patients in the two arms, there is evidence that DAIR requires a lesser invasive procedure compared to a one-stage revision, delivers a higher functional knee score, and enhances recovery [2, 6–8].

A large observational study or randomized controlled trial by the infection-specific surveillance networks involving multiple sites and countries is important to provide a high level of evidence on the criteria to perform DAIR [10]. Greater research funding should be provided to identify and implement cost-effective arthroplasty-related PJI prevention and management interventions [11].

This study has some limitations. It used data from public hospitals in a single state in Australia. In Queensland, the majority of arthroplasty surgeries are performed in the private setting, while a few arthroplasties are performed in public hospitals. The use of data that represents the state’s or national population would have provided a more robust result. Second, the sample size for DAIR was relatively small compared to one-stage revision, so results between the two groups should be interpreted with caution. The reason for the small sample size was the stringent inclusion criteria, which aimed to minimize analytical bias. There is a possibility that the study may not have adequately controlled for all compelling indications for a staged revision due to a lack of a clearly defined protocol for DAIR management. Further research is required to overcome this limitation. Future research should also consider a comprehensive economic evaluation of DAIR versus a one-stage revision with more stringent criteria in the selection of patients for DAIR.

## Conclusion

Based on available evidence, this study suggests the use of one-stage septic revision over DAIR for acute postoperative PJI and acute hematogenous infection, where there is no compelling indication for a staged revision. It suggests that one-stage revision should be given priority over DAIR to minimize the risk of re-revision, unless when the treating medical team deems it fit to perform DAIR. Further research on the use of DAIR is required to provide a well-defined treatment protocol with a high level of evidence. Treatment should always be performed within a multi-disciplinary team setting with evidence-based advice from all experts in the team.

## Supplementary Information

Below is the link to the electronic supplementary material.

Supplementary file1 (XLSM 54 KB)

## Data Availability

The data used for this analysis has been provided as an electronic supplementary file during the review process. Individual patient level data are available upon request.
